# Extracellular Matrix Hydrogels Originated from Different Organs Mediate Tissue-Specific Properties and Function

**DOI:** 10.3390/ijms222111624

**Published:** 2021-10-27

**Authors:** Tzila Davidov, Yael Efraim, Rotem Hayam, Jacopo Oieni, Limor Baruch, Marcelle Machluf

**Affiliations:** Faculty of Biotechnology & Food Engineering, Technion—Israel Institute of Technology, Haifa 3200003, Israel; tzila937@gmail.com (T.D.); yael.efraim@gmail.com (Y.E.); rotemh27@gmail.com (R.H.); jacopo.oieni@gmail.com (J.O.); baruchl@bfe.technion.ac.il (L.B.)

**Keywords:** extracellular matrix, tissue-specific, hiPSC, ECM hydrogel

## Abstract

Porcine extracellular matrix (pECM)-derived hydrogels were introduced, in recent years, aiming to benefit the pECM’s microstructure and bioactivity, while controlling the biomaterial’s physical and mechanical properties. The use of pECM from different tissues, however, offers tissue-specific features that can better serve different applications. In this study, pECM hydrogels derived from cardiac, artery, pancreas, and adipose tissues were compared in terms of composition, structure, and mechanical properties. While major similarities were demonstrated between all the pECM hydrogels, their distinctive attributes were also identified, and their substantial effects on cell-ECM interactions were revealed. Furthermore, through comprehensive protein and gene expression analyses, we show, for the first time, that each pECM hydrogel supports the spontaneous differentiation of induced pluripotent stem cells towards the resident cells of its origin tissue. These findings imply that the origin of ECM should be carefully considered when designing a biomedical platform, to achieve a maximal bioactive impact.

## 1. Introduction

Accumulating knowledge on the extracellular matrix (ECM) interactions with the tissue and its cellular components has revealed the major role of tissue ECM in regulating cellular processes in health and disease, as well as in governing cell behavior and fate [1,2]. This had led, in recent years, to the development of in vitro and in vivo biomedical applications using tissue-derived ECM, thus aiming to achieve a physiological-like cell behavior in addition to improved cell survival [3].

The tissue ECM consists of two main classes of macromolecules: fibrous proteins, including collagen and elastin; and glycoproteins, including proteoglycans, fibronectin, and laminin [4,5,6]. This combination of macromolecules generates a fibrous microstructure that grants the tissues the mechanical and physical properties needed for their function. Furthermore, the ECM is responsible for transmitting a wealth of chemical and mechanical signals that mediate key processes of cellular physiology such as adhesion, migration, proliferation, differentiation, and death [7]. The ECM of each tissue is produced by its residing cells through an interplay between the different cell types, which secrete molecules affecting the distinctive 3D organization and biochemical profile of the tissue, providing the ideal microenvironment for their function [7]. The ECM of different tissues is, consequently, characterized by a different tissue-specific composition, organization, and structure [8].

Using ECM as a biomaterial in biomedical platforms can, therefore, harness its tissue-specific microstructure and bioactivity to promote regenerative processes and cellular functions [8,9,10]. Nevertheless, like all natural materials, the use of ECM holds several limitations, such as batch-to-batch variability, limited control over the construct’s size and structural features, and limited delivery options [8]. To generate a more uniform product that also enables the incorporation of other materials and entities such as drug delivery systems, the processing of ECM into a hydrogel was suggested, thus opening the gate to implementing ECM in a whole new range of applications [11,12]. Moreover, the use of ECM in its hydrogel form allows its molding or 3D-bioprinting into different shapes and patterns as well as injecting it through minimally invasive devices.

We, therefore, hypothesized that the tissue-specific characteristics of each ECM will significantly affect its function as a biomaterial when processed into a hydrogel for different biomedical applications, both in terms of the structural and mechanical properties of the hydrogel and in terms of its interplay with the residing cells, i.e., its ability to support their viability and function as well as to direct stem cells differentiation toward the tissue from which the ECM was originated.

In the current work, we, therefore, addressed the different characteristics of porcine ECM (pECM) hydrogels derived from different tissues, in terms of composition, structure, and mechanical properties and studied their effects on cell culture and differentiation through total RNA sequencing analyses.

## 2. Results

### 2.1. pECM Hydrogels Originating from Different Tissues Have Distinctive Composition

ECM hydrogels were produced from porcine heart, pancreas, aorta, and adipose tissue using solubilization protocols that were optimized to result in stable pECM hydrogels, suitable for in vitro and in vivo applications (Table 1, Section 4).

**Figure 1:**
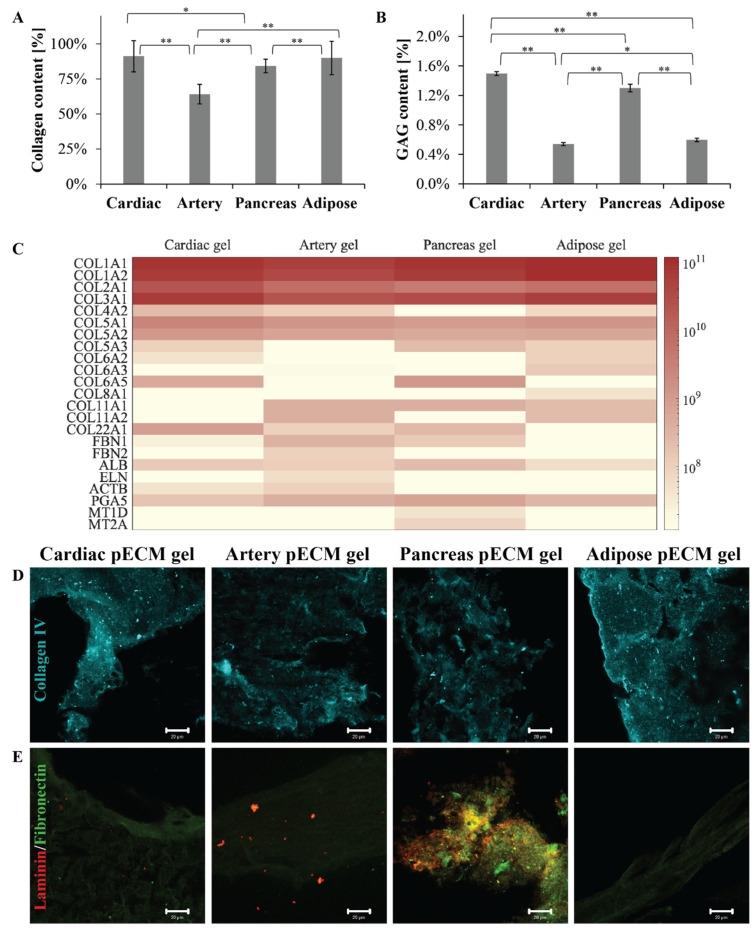
pECM hydrogels composition. (**A**) Collagen and (**B**) GAGs quantification in the different pECM hydrogels, presented as a percentage of total dry pECM of the hydrogels. * *p* < 0.05, ** *p* < 0.01 (**C**) Proteomic profile of the different pECM hydrogels presented as a heatmap for each hydrogel. The scale ranges from white to maroon where maroon represents a more abundant protein, according to LFQ calculated parameter. (**D**,**E**) Immunostaining of the different pECM hydrogels for collagen IV (**D**, cyan), laminin, and fibronectin (**E**, red and green, respectively). Scale bars, 20μm.

To compare the different pECM hydrogels, we primarily addressed their composition, thus quantifying their most abundant components: collagen and glycosaminoglycans (GAGs). As seen in Figure 1A, all the pECM hydrogels were mainly composed of collagen. Nevertheless, while the arterial pECM hydrogel contained nearly 64% collagen, the collagen content of cardiac, pancreas, and adipose pECM hydrogels was more than 20% higher, ranging between 84% and 91% (*p* < 0.01). GAGs content was significantly (*p* < 0.01) higher in the cardiac and pancreas pECM hydrogels (1.50 ± 0.03% and 1.30 ± 0.05%, respectively), compared to the adipose and artery pECM-derived hydrogels (0.60 ± 0.02% and 0.54 ± 0.02%, respectively, Figure 1B). To further address the pECM hydrogels protein composition, a proteomic analysis was conducted, emphasizing that all pECM hydrogels mostly consist of collagen types I, II, and III, with slight variation in their prevalence in each hydrogel (Figure 1C). In particular, a higher content of collagen type I characterized the adipose pECM hydrogel. In the less abundant proteins, however, a unique profile was revealed for each pECM hydrogel. For example, artery pECM hydrogel is the only hydrogel that contains elastin and fibrillin. In the cardiac pECM hydrogel, on the other hand, higher quantities of collagen types V and XXII were detected while collagen type XI was absent. Intriguingly, no collagen type IV was detected in the pancreas pECM hydrogel, though pancreatic ECM is known to contain significant levels of this collagen [13]. Similarly, in none of the hydrogels, we could detect fibronectin or laminin, known ECM components. To test whether these proteins were lost during the hydrogel preparation or whether the proteomic analysis failed to detect them, we further performed immunostaining analyses. These analyses confirmed that all the pECM hydrogels contained collagen type IV (Figure 1D). Fibronectin, on the other hand, was present in the pancreas pECM hydrogel to a significantly larger extent than in the other hydrogels, and laminin could only be detected in the pancreas and artery pECM hydrogels (Figure 1E).

**Figure 2:**
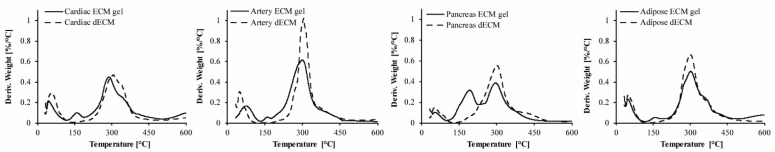
Preservation of the pECM hydrogels composition. Thermal gravimetric analysis of pECM hydrogels and decellularized pECM. TG 1st derivative curve of cardiac, artery, pancreas, and adipose pECM.

To evaluate the preservation of the pECM components through its processing into hydrogels, we performed thermal gravimetric analysis (TGA) and compared the different decellularized ECMs (dECM) to their respective hydrogels [14,15]. As seen in Figure 2, Table 2, and Figure S1, three degradation steps are obtained for all the pECM samples. The first degradation process starts below 50 °C and is attributed to the loss of bound water. The second degradation process in the hydrogels is seen at ~130 °C and is attributed to the degradation of pepsin, the enzyme that is used for pECM solubilization. The third process that starts at ~230 °C is the most dominant one and is attributed to the degradation of the collagenous components [15]. Overall, our results show minor differences between each hydrogel and the dECM from which it originated, except for the additional step of pepsin degradation. Weight loss derivative analyses enabled determining for each degradation process the initial temperature of the thermal degradation (Tonset), the maximal temperature of the thermal degradation (Tpeak), the temperature in which the degradation was completed (Tendset), and the weight percentage loss during each stage (W). When comparing the different pECM hydrogels, similar thermal decomposition processes were observed, showing residual material below 30%. The second degradation process was the most dominant in the pancreas hydrogel, due to the higher pepsin concentration required for the solubilization of this pECM. For the third degradation process, an earlier onset was seen in the artery pECM hydrogel compared to the pancreas accompanied by a lower endset temperature, compared to the cardiac hydrogel. A higher residue was obtained for the pancreas hydrogel compared to the other pECM hydrogels.

### 2.2. pECM Hydrogels Originating from Different Tissues Are Characterized by Unique Structures and Mechanical Properties

**Figure 3:**
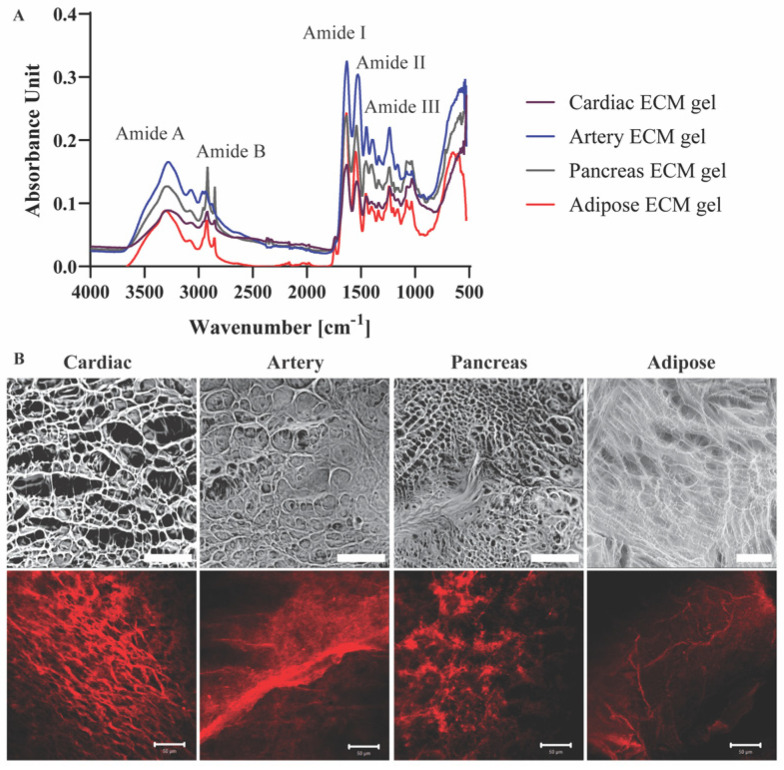
pECM hydrogels structural characterization. (**A**) FTIR spectra of pECM hydrogels. (**B**) Upper panel: SEM images of pECM hydrogels. Scale bar, 100 μm. Bottom panel: Immunostaining of collagen I. Scale bars, 50 μm.

Molecular structures and the presence of characteristic groups, such as amide bonds, sulfates, and sugars in the different pECM hydrogels were addressed using FTIR analyses [14]. The four pECM hydrogels revealed amide vibrations proximal to amide A (~3300 cm^−1^), amid B (~3100 cm^−1^), and the three main bands of the collagen fingerprint: amide I (~1650 cm^−1^) due to carbonyl stretching, amide II (~1550 cm^−1^) due to combination of N-H in-plane bend, and C-N stretching vibrations, and amide III (~1200 cm^−1^) due to combination of the C-N stretching vibrations and N-H bending (Figure 3A) [16,17,18,19]. Though no significant differences are evident between each hydrogel and its native dECM (Figure S2), pECM hydrogels derived from different tissues vary in the relative amount of each band, as can be seen from the different peak absorbance ratios (Figure 3A).

To assess the three-dimensional microstructure of the pECM hydrogels, collagen I immunostaining and SEM analyses were performed, showing a different rearrangement of the collagen fibers in each hydrogel. In particular, while the collagen fibers of the cardiac, artery and pancreas pECM hydrogels had a relatively homogenous size distribution, in the adipose pECM hydrogel, extremely thin collagen fibers branched from significantly thicker ones. These analyses revealed that while all hydrogels are characterized with a porous isotropic net structure, each hydrogel exhibits a unique pattern, differing in fiber diameter, fiber diameter distribution, and pore size (Figure 3B).

**Figure 4:**
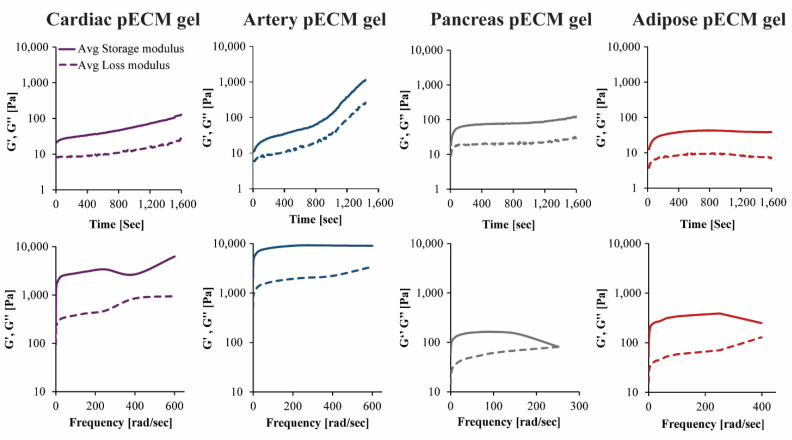
Mechanical properties of the pECM hydrogels. Upper panel: Time sweep rheological characterization, changes in storage (G’) and loss (G’’) moduli over time. Lower panel: Frequency sweep rheological characterization, changes in storage (G’) and loss (G’’) moduli in ascending frequencies.

The rheological properties of the different pECM hydrogels were characterized using time and frequency sweep analyses. In the time sweep analyses, measurements were taken while rising the temperature from 4 °C to 37 °C, to enable gelation. Both storage modulus (G′) and loss modulus (G′′) had changed over time. In the hydrogels originating from the pancreas and adipose pECM, gelation had occurred after approximately 4 min, when G′ and G′′ reached a steady state. In the hydrogel originating from cardiac and artery pECM, however, the storage modulus kept rising and did not reach a steady state by the end of the analysis (Figure 4).

In the frequency sweep analysis, hydrogels were exposed to small deformation oscillations covering a range of frequencies to assess the structural response to deformations with longer or shorter timescales. In all pECM hydrogels, the storage modulus was higher than the loss modulus over the entire frequency range, and both moduli were only slightly dependent on frequency, thus indicating a weak gel behavior [20]. As the deformation increased, the hydrogels underwent a progressive breakdown of the three-dimensional network. As seen in Figure 4, the artery pECM hydrogel was the strongest hydrogel, thus exhibiting the highest storage modulus and a moderate deformation point at 380 rad s^−1^. The weakest hydrogel, on the other hand, was the pancreas pECM, which had the lowest storage modulus and a deformation point at approximately 160 rad s^−1^.

### 2.3. Culturing Stem Cells on pECM Hydrogels

**Figure 5:**
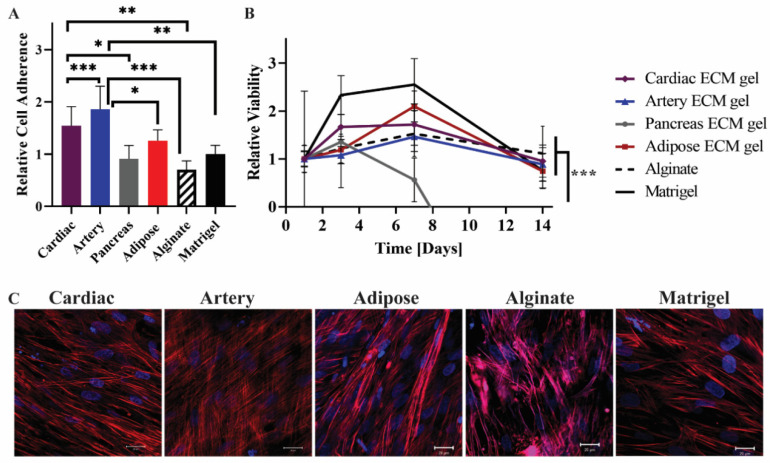
MSCs cultivation on the pECM hydrogels. (**A**) Cell adherence to the pECM hydrogels, and (**B**) viability over 14 days, relative to the viability on each material at day 1 (control). n = 5, * *p* < 0.05, ** *p* < 0.01, *** *p* < 0.0001. (**C**) Fluorescent microscope images of MSCs on the hydrogels 14 days post-seeding. Red: Phalloidin (Actin), Blue: DAPI (DNA). Scale bars, 20 µm.

To study the interactions of mesenchymal stem cells (MSCs) with pECM hydrogels from different tissues, we assessed the adhesion, viability, and morphology of the MSCs cultured on the pECM hydrogels. As a control for these experiments, we used alginate and matrigel, common support materials used for cell culture, and tissue engineering scaffolds [21,22,23]. As can be seen from the relative viability detected on the first day, all the hydrogels supported the adherence of MSCs at different levels (Figure 5A). Along the first 7 days of culture, an increase in cell number was observed, leading to constant viability levels until day 14 on most of the pECM hydrogels. On the pancreas pECM hydrogel, however, a decrease in MSCs’ viability was observed until day 7, when almost no viable cells were detected (Figure 5B). Actin staining analysis of the MSCs following 14 days of culture, revealed elongated morphology of the cells under confocal microscopy. Nevertheless, different cell alignment patterns were observed, such as alignment to the same direction in a tissue-like structure on the cardiac pECM hydrogel, a semi-aligned morphology on the adipose tissue pECM hydrogel and the matrigel, and a mesh structure on the artery pECM hydrogel (Figure 5C).

### 2.4. pECM Hydrogels Induce Spontaneous Differentiation of hiPSCs

The effect of pECM from different tissues on the culture and differentiation of hiPSCs was studied through the culturing of cells on the different hydrogels. To allow gel stability along the 28 days of hiPSCs culture and differentiation, the hydrogels had to be crosslinked using natural collagen crosslinkers [9,24,25]. The crosslinking of the different pECM hydrogels was, therefore, evaluated in terms of the adherence of hiPSCs (Figure S3), and the most appropriate crosslinking regimes were chosen as follows: for the cardiac, pancreas, and adipose pECM hydrogels 0.1% genipin, and for the artery pECM hydrogel 0.1% epigallocatechin gallate (EGCG).

**Figure 6:**
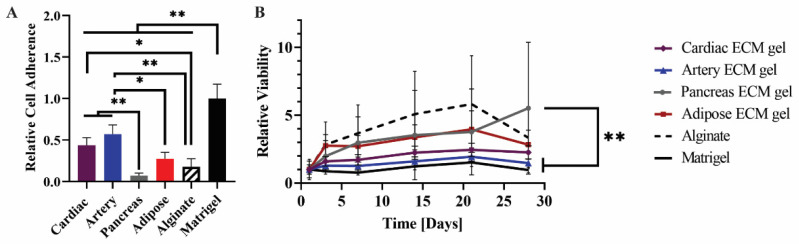
hiPSCs culture on the pECM hydrogels. (**A**) Cell adherence on the pECM hydrogels, and (**B**) viability along 28 days of spontaneous differentiation on different pECM hydrogels relative to the viability on each material at day 1 (control). n = 5, * *p* < 0.01, ** *p* < 0.001.

**Figure 7:**
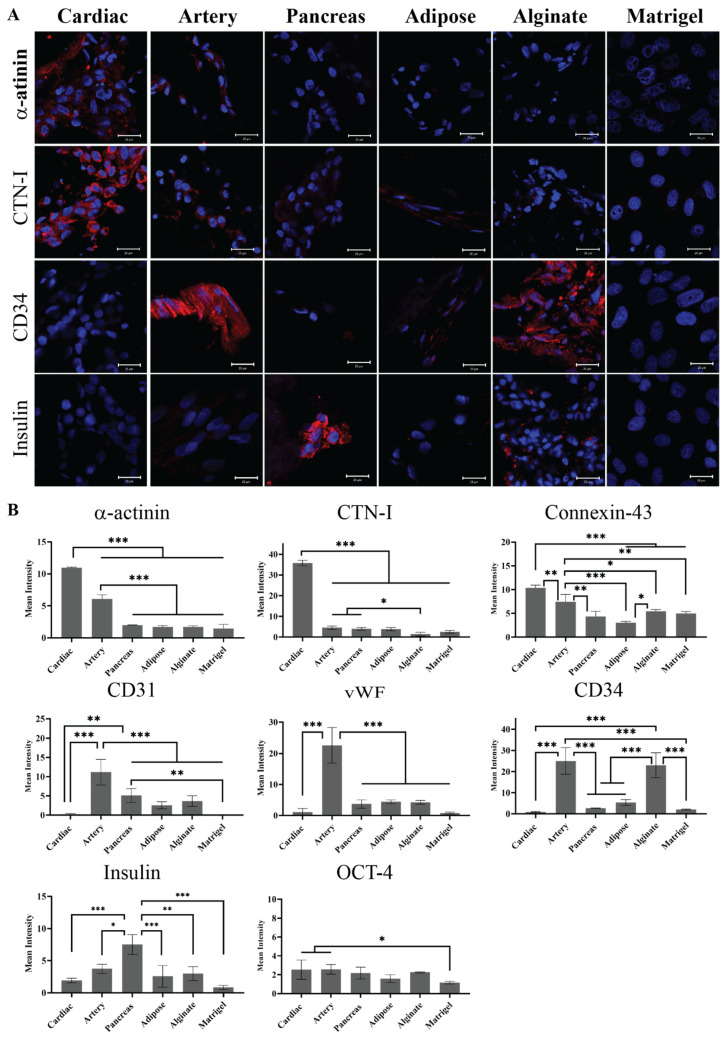
Spontaneous differentiation of hiPSCs on pECM hydrogels. (**A**) Immunostaining of spontaneously differentiated hiPSCs 28 days post-seeding. Red: α-actinin, cTnI, CD34, and Insulin. Blue: DAPI (DNA). Scale bars, 20 µm. (**B**) Quantification is expressed as the intensity of immunostained markers normalized to the number of cells. * *p* < 0.05, ** *p* < 0.01, *** *p* < 0.001.

The hiPSCs were then seeded on the different pECM hydrogels as well as matrigel and alginate controls. Pluripotency maintenance media was then removed and replaced with media that is known to induce iPSCs spontaneous differentiation, as defined in the Section 4 [26]. As seen in Figure 6A, different adherence rates were obtained on the different hydrogels with the highest rate of adherence to the matrigel and the lowest rate of adherence to the pancreas pECM hydrogel (*p* < 0.0001). Further, the hiPSCs remained viable on all the hydrogels for at least 28 days (Figure 6B). We then evaluated the expression of markers that are specific for cells residing in the tissues from which the pECMs were originated, to examine whether hiPSCs cultured on the different pECM hydrogels had spontaneously differentiated towards these lineages. Positive immunostaining for the cardiac markers, α-actinin and cardiac troponin I (CTN-I), was observed in the hiPSCs seeded on the cardiac pECM hydrogel and at a lower expression level on the artery pECM hydrogel, but not on the other hydrogels. Similarly, the connexin-43 cardiac marker was mainly stained in the cardiac and artery pECM hydrogels. The endothelial markers CD34, CD31, and Von Willebrand factor (vWF) were all expressed on the artery pECM hydrogel, with lower levels of CD31 expression on the pancreas, adipose, and alginate hydrogels, and a similar level of CD34 expression on the alginate. Insulin was mainly expressed on the pancreas pECM hydrogel (*p* < 0.001, Figure 7, Figure S4).

**Figure 8:**
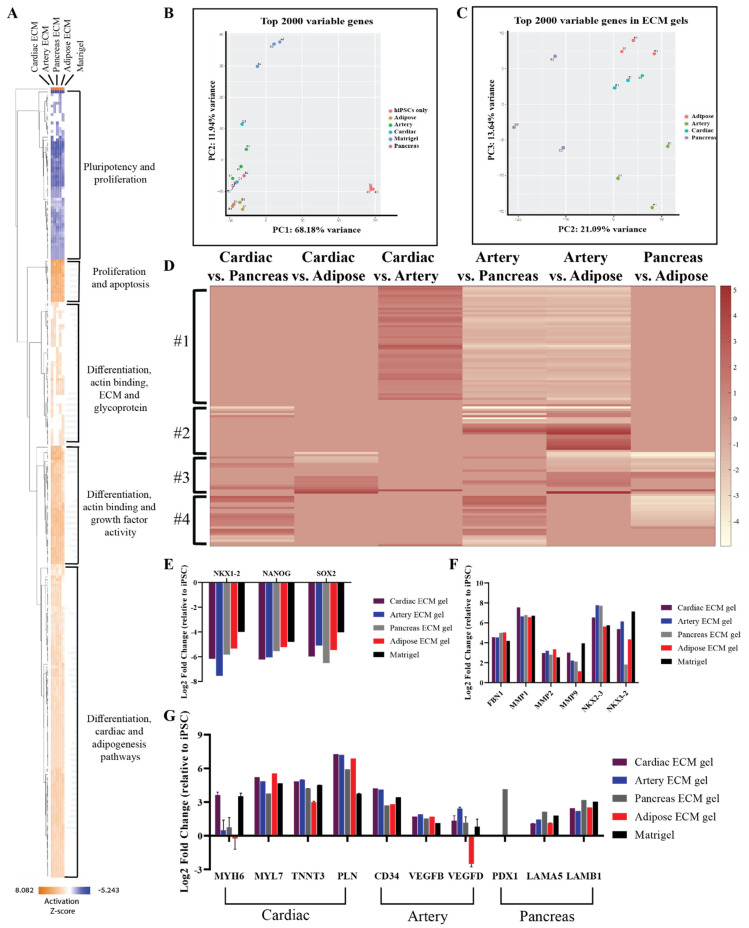
RNA-seq of spontaneously differentiated hiPSCs. (**A**) Hierarchical clustering heat map of gene expression by hiPSCs cultured on the different pECM hydrogels and matrigel relative to undifferentiated hiPSCs. Relative expression (z-score) is displayed in colors, in which orange indicates upregulation and blue indicates downregulation. (**B**,**C**) Principal component analysis (PCA) of the top 2000 variable genes. (**B**) A comparison between cells cultured on all pECM hydrogels, matrigel, and undifferentiated hiPSCs control. (**C**) A comparison between cells cultured on the pECM hydrogels. (**D**) Heat map of relative gene expression (z-score) by hiPSCs cultured on the different pECM hydrogels. Maroon indicates upregulation and white indicates downregulation. (**E**,**G**) Expression of selected genes relative to undifferentiated hiPSCs. (**E**) Genes associated with pluripotency: NKX 1-2, NANOG, SOX-2. (**F**) Genes associated with ECM and ECM remodeling: Fibronectin 1, MMP1, MMP2, MMP9, NKX 2-3, NKX 3-2. (**G**) Specific tissue differentiation: MYH6, MYH7, TNNT3, PLN, CD34, VEGFB, VEGFD, PDX1, LAMA5, and LAMA6.

To further confirm the tissue-specific spontaneous differentiation of hiPSCs that was observed in the immunostaining analyses and to reveal other possible effects of the different pECM hydrogels, comprehensive gene expression analyses were performed (Figure 8). Gene profile comparison to the undifferentiated hiPSCs, show the initiation of differentiation processes of the cells cultured on the pECM hydrogels, where pluripotency-related genes were downregulated, and proliferation and differentiation genes were upregulated as seen in the hierarchical clustering heat map (Figure 8A). These results were emphasized in the principal component analysis (PCA, Figure 8B), which revealed the major differences between the gene expression profile of undifferentiated hiPSCs to the ones of hiPSCs that were cultured on the different hydrogels. Further, this analysis clearly showed that cells cultured on matrigel had a significantly different expression profile from that of the cells cultured on the pECM hydrogels (Figure S5). One significant difference was the upregulation of a cluster of tumor-associated genes on the matrigel. When focusing solely on the pECM hydrogels-cultured hiPSCs, the tissue-specific processes are evident through the separated clusters in the PCA and the differential gene expression profiles (Figure 8B–D). These profiles showed a specific gene cluster that was downregulated in the artery pECM hydrogel compared to other pECM hydrogels (Figure 8D, #1). In another cluster, genes’ expression differed between the artery pECM hydrogel and the adipose one, but only partially differed between the artery and the pancreas one and was not altered in the cardiac ECM hydrogel (Figure 8D, #2). These genes were mostly related to the family of transcription factors involved in the regulation of embryonic development, TnI-cardiac protein that is expressed in cardiac muscles, and transcription factor that regulates mesenchymal-epithelial communication. The expression of genes in the third cluster was particularly different on the adipose pECM hydrogel, of which upregulation of glycoproteins as well as of genes that are typically expressed in adipose tissue was obtained, such as CCDC80, CDC42EP3, and GNAI1 that are related to the family of guanosine triphosphate (GTP). They regulate actin cytoskeleton re-organization during cell shape changes and are part of a complex that responds to beta-adrenergic signals (Figure 8D, #3). In the fourth cluster, unique expression patterns characterized the pancreas pECM hydrogel (Figure 8D, #4).

Examining the expression of specific genes, a decrease in pluripotency genes such as NKX1-2, NANOG, and SOX-2 was evident on all the hydrogels (Figure 8E). Also, there was an increase of ECM-associated genes and Matrix metallopeptidases (MMP), such as fibronectin, MMP1, MMP2, and MMP9 (Figure 8F). The cardiac differentiation-associated genes, myosin heavy chain, troponin, and GATA-4 reached the highest expression levels on cardiac and artery pECM hydrogels, while the expression of myosin light chain was the highest on the adipose ECM hydrogel. Among the genes associated with blood vessel formation, the expression of CD34 was higher on the cardiac and artery pECM hydrogels and the expression of vascular endothelial growth factor D (VEGFD) was higher on the artery pECM hydrogel. PDX-1 gene, which is strongly associated with differentiation towards pancreatic lineages, was only upregulated on the pancreas pECM hydrogel. Furthermore, laminin genes expression was the highest on this hydrogel (Figure 8G).

## 3. Discussion

Decellularized ECM-based hydrogels raise a growing interest as a biomaterial for regenerative medicine [11,27,28]. In addition to mimicking the physiological environment, thus granting their residing cells with their native conditions, ECM hydrogels can also be tailored into diverse structures and physical properties that benefit their use for multiple biomedical applications [8,11]. Nevertheless, many issues related to the use of ECM hydrogels remain to be verified, such as the effect of ECM origin on the physical and―most importantly—the biological attributes of the resulting hydrogel.

To address this issue, we have produced four hydrogels from porcine ECM, which were isolated from four different tissues that are commonly investigated for regenerative therapy: cardiac, pancreas, artery, and adipose tissue [7,29]. Due to the particular properties of pECM of different tissues, each hydrogel was produced using the minimal ECM concentration that allowed its self-assembly into a hydrogel at 37 °C. These concentrations (Table 1) resulted in stable hydrogels that can be readily applied to in vitro and in vivo applications, corresponding with the commonly reported ECM and collagen-based hydrogels of 1–60 mg/mL, applied as hydrogel scaffolds, coating layers, or printable bioinks [10,30,31,32,33,34].

In terms of pECM hydrogel composition, collagens are known to be the main component of all ECMs [6,35]. Our results showed that while pECM hydrogels from the cardiac, pancreas, and adipose tissue were composed of 80% to 90% collagen, the artery pECM hydrogel contained a lower amount of collagen, probably due to a significant elastin content, as previously suggested [29,36]. Furthermore, each hydrogel exhibited a unique tissue-specific profile of different collagens that was previously shown to represent the molecular milieu of the original tissue ECM [29,37]. The most abundant collagens were collagen I and III in all the hydrogels, and the less abundant collagens were only identified in some of them. This tissue-specific profile corresponds to earlier publications, showing that also in human ECM, the distribution of collagens and glycoproteins such as elastin, fibrillin, laminin, and others differs according to the source organ [38,39] and is of high relevance to the tissue’s function [27]. Surprised by the absence of particular proteins we expected to find in the pECM hydrogels, we performed immunostaining analyses, which revealed the presence of collagen type IV in all the pECM hydrogels. Fibronectin was mainly found in the pancreas pECM hydrogel but also, to different extents, in the other hydrogels. Laminin, on the other hand, is expected in all the tissues’ ECM but was hardly found in the cardiac and adipose tissue pECM hydrogels. The differences in the ECM components can also be attributed to the decellularization method. Nevertheless, avoiding the use of SDS, which was found to reduce or remove GAGs and particularly Laminin, [40] could have contributed to the preservation of these components in the ECM of tissues where they are more abundant, thus maintaining a unique molecular fingerprint of each tissue-derived pECM hydrogel [13,41,42].

Due to their mechanical and structural contributions, glycosaminoglycans are an important non-protein component of ECM, essential to tissue regeneration [6,43]. Similar to our previous publications, [9,37] the GAGs mass percentage was between 0.5–1.5% of the hydrogels.

The preservation of pECM components in the respective hydrogels was addressed through their thermal degradation processes, which were generally similar between the pECM hydrogels and their original tissue ECM. In the weight derivative analysis, however, additional peaks were observed in the hydrogels that corresponded to the pepsin used for their solubilization. In terms of molecular structure, FTIR analysis has shown the amide vibration profile in all pECM hydrogels typical for collagen-based materials. Furthermore, the different amide vibrations were preserved through the hydrogel preparation process for all hydrogels. Addressing the microstructure of the pECM hydrogels, we could see that while all hydrogels had a fibrous collagenous structure, the size and density of the fibers in each hydrogel are distinct, hence the unique composition of each tissue’s ECM dictates the unique microstructure of each hydrogel [44,45].

Rheological analyses demonstrated a consistently greater storage modulus than loss modulus values for all the pECM hydrogels, typical for weak gels that undergo a progressive breakdown in the three-dimensional networks as the deformation increases [20]. Nevertheless, a tissue-specific rheological behavior was seen for the different pECM hydrogels, demonstrated through the different deformation points and the different values of storage and loss moduli, thus distinguishing between the stronger pECM hydrogels (i.e., artery and cardiac) and the weaker ones (i.e., pancreas and adipose) regardless of their concentrations. For example, while both cardiac and adipose pECM hydrogels comprised of 10% pECM, the cardiac pECM hydrogel had higher storage and loss modulus, and higher breaking point than the adipose pECM hydrogel. Moreover, the pancreas pECM hydrogel, which is comprised of 20% pECM had the lowest storage and loss modulus and the lowest breaking point.

Aiming to better understand the implications of using pECM hydrogels from different tissues for biomedical applications, we studied cell-ECM interactions on the different pECM hydrogels. Initially, we have addressed the cultivation of MSCs, which are commonly used in regenerative therapy research as well as in cell-based therapies [38,46,47]. The adherence of the MSCs was dependent on the origin of the pECM hydrogel, however, cell proliferation was generally similar on all the hydrogels except the pancreas one, where their viability decreased to zero along 7 days. Stem cells’ behavior is known to be affected by the mechanical properties and microstructure of their surrounding ECM, which in turn depends on the molecular composition and organization [48]. Through mechanical signaling, the elasticity of the hydrogel can thus affect cell adherence, morphology, proliferation, and fate [49,50]. Our results showed that the pancreas pECM hydrogel, which was characterized by the lowest storage modulus and the lowest deformation point did not support MSCs’ culture, possibly due to insufficient mechanical resistance [51,52]. On the other hand, the relatively small pore size of the pancreas pECM hydrogel could also contribute to its failure to support MSCs’ culture [53,54].

The different cellular alignment patterns obtained on the different hydrogels, also suggest that a distinctive microenvironment is obtained depending on the tissue of origin [48,50]. To further investigate whether a tissue-specific microenvironment is provided to resident cells by the pECM hydrogels, we addressed the effect of the different hydrogels on stem cell differentiation.

Holding the potential for an unlimited supply of autologous stem cells, hiPSCs were suggested as a viable option for stem cell transplantation in many regenerative therapies [46]. We, therefore, examined their spontaneous differentiation when seeded on pECM hydrogels derived from different tissues. Nonetheless, to allow gel stability along the 28 days of hiPSCs culture and differentiation, the mechanical properties of the hydrogels had to be adapted through the addition of crosslinkers. To this end, we evaluated the adherence of hiPSCs to hydrogels supplemented with the native protein crosslinkers, genipin and EGCG, [24,25,45] and chose the most appropriate combination for the differentiation studies. When culturing hiPSCs on the pECM hydrogels, different adherence and proliferation levels were obtained, as the hiPSCs are influenced by their immediate surrounding containing specific molecules for their growth and differentiation [55]. However, all the hydrogels supported hiPSCs culture for at least 28 days. The spontaneous differentiation of hiPSCs cultured on pECM hydrogels derived from different tissues was initially addressed using immunostaining of markers specific to cells residing in the tissues of origin. Strikingly, these analyses indicated that each pECM hydrogel had directed his residing hiPSCs’ differentiation towards the lineages typical of the pECM original tissue. These results inspired us to take a deeper dive into the effect of pECM hydrogels on hiPSCs’ tissue-specific spontaneous differentiation, and further address it using gene expression sequencing analyses. The differentiated hiPSCs were analyzed using the Cell expression by linear amplification and sequencing (CEL-Seq) method. This method is a linear amplification in vitro transcription method, using barcoding for efficient analysis of multiple samples at the same time, [56] to evaluate the unique gene expression profile of each sample. Primarily, we noted that gene expression profiles obtained from hiPSCs cultured on the hydrogels were significantly different from the ones of hiPSCs cultured on matrigel, or the ones of the non-differentiated hiPSCs. Hence, spontaneously differentiated hiPSCs showed higher expression levels of differentiation-related genes and apoptosis-related genes, while the undifferentiated cells showed higher expression levels of pluripotency and proliferation markers. When comparing hiPSCs differentiated on pECM hydrogels to the ones differentiated on matrigel it is evident that cytokine activity and differentiation genes were upregulated on the ECM hydrogels, while apoptosis- and tumor-associated genes were upregulated on the matrigel. This is probably due to the cancerous origin of matrigel―mouse sarcoma cells [57]. When focusing solely on the tissue-derived pECM hydrogels, the unique tissue-specific behavior was even more conspicuous, as each pECM was seen in a distinct PCA cluster. Notably, in some cases, differences were seen between the patterns of RNA and protein expression of the same genes. For example, similar RNA levels of CD34 were obtained on the cardiac and artery-derived ECM hydrogels, whereas notable protein expression was only obtained on the artery ECM hydrogel. This can be explained by the regulation of protein translation in the cell [58].

Overall, our results demonstrate the critical role of the tissue from which an ECM hydrogel is derived in directing stem cell differentiation. These results correspond with previous studies that demonstrated the beneficial effect of a tissue-specific microenvironment achieved using solid, [59] soluble, [60] or hydrogel [10,61] ECM on its residing cells. Furthermore, the use of different forms of ECM—but not hydrogel—was demonstrated to enhance tissue-specific differentiation mainly in the context of osteogenic stem cell differentiation, [44,59,62] but also in neuronal differentiation [28]. Specifically, Beachley et al. had revealed a different response of the cells when exposed to ECM of different origins, and even a correlation between the chondrocyte and osteoblast differentiation to specific components of particular tissue-derived ECM [59]. This previous research supports our findings that the hydrogels derived from ECM of particular tissues preserve the tissue-specific composition and unique structural features of the ECM. Consequently, they also preserve their tissue-specific biological attributes, allowing active guidance of the spontaneous differentiation of non-committed pluripotent stem cells towards different lineages that characterize each ECM’s tissue of origin.

## 4. Materials and Methods

### 4.1. pECM Decellularization

pECM was decellularized and sterilized according to our previously published protocols for the cardiac, artery, and pancreas tissues [29,37,63], (patent US9216236B2) Briefly, left ventricular tissue, pancreas, and aorta were isolated from healthy commercial slaughter-weight pigs purchased from LAHAV C.R.O’s slaughterhouse (Lahav, Israel). The decellularization procedure consisted of two cycles of the following steps: Alternating hyper/hypotonic NaCl solutions, enzymatic treatment using trypsin (Sigma-Aldrich, St. Louis, MO, USA) and detergent washes with Triton-X-100 (Merck Millipore, Burlington, MA, USA).

Porcine retroperitoneal adipose tissue was obtained from the Technion pre-clinical research authority and decellularized using three 30 min cycles of freezing and thawing, enzymatic treatment using trypsin, and detergent washes with Triton-X-100.

### 4.2. Hydrogel Preparation

The decellularized pECM was frozen in liquid nitrogen, crushed using mortar and pestle, and lyophilized to a dry fine powder. To obtain stable pECM hydrogels, dry pECM at concentrations, as detailed in Table 1, was solubilized in HCl (0.01 M) using 1 min sonication, followed by enzymatic digestion using pepsin (1-5 mg ml^−1^, Sigma-Aldrich, St. Louis, MO, USA). The pH of the solution was then elevated using NaOH and kept cold (4 °C), as previously described [9,63,64]. To allow the thermally-induced self-assembly of the hydrogels, solubilized samples were plated for 1 hr at 37 °C. Subsequently, PBS was added to prevent dehydration.

The Alginate hydrogel was produced as followed: 1% (*w*/*v*) aqueous solution sodium alginate (LVG, NovaMatrix, Sandvika, Norway) was mixed with calcium chloride solution (0.03% *w*/*v*, CaCl_2_, Merck Millipore, Burlington, MA, USA).

### 4.3. Composition Analyses

The total collagen and GAGs content in the pECM hydrogels was quantified using a Sirius red assay, and Safranin-O method, respectively, as previously described [65,66].

### 4.4. Proteomic Analysis

Proteomic analysis was performed at The Smoler Protein Research Center, Technion –Israel Institute of Technology, Haifa, Israel. Samples were digested by trypsin and the resulting peptides were analyzed by mass spectrometry using Q-Exactive plus mass spectrometer (Thermo Fisher Scientific, Waltham, MA, USA) in a positive mode using repetitively full MS scan followed by collision induces dissociation (HCD) of the 10 most dominant ions selected from the first MS scan. The mass spectrometry data was analyzed using the MaxQuant software 1.5.2.8 (Max-Planck-Institute of Biochemistry, Planegg, Germany) for peak picking and identification using the Andromeda search engine, searching against the sus proteome from the Uniprot database with mass tolerance of 6 ppm for the precursor masses and 20 ppm for the fragment ions. Oxidation on methionine and protein N-terminus acetylation were accepted as variable modifications and carbamidomethyl on cysteine was accepted as static modifications. Minimal peptide length was set to six amino acids and a maximum of two miscleavages was allowed. The data was quantified by label free analysis using the same software. Peptide- and protein-level false discovery rates (FDRs) were filtered to 1% using the target-decoy strategy. The obtained data is presented in the form of a heatmap for log2 of protein abundance in each sample, which was calculated according to the Label-Free Quantification (LFQ) parameter.

### 4.5. Immunofluorescent & Fluorescent Staining

Samples were fixed in paraformaldehyde (PFA, 4%) for 20 min, washed in PBS, frozen in Tissue-Tek^®^ OCT compound (Sakura, Netherlands), and cross-sectioned using a cryostat (Leica Microsystems, Wetzlar, Germany) into slices (10 μm) on glass slides for staining. Slides were fixed in cold MeOH (4 °C) for 20 min before staining. After fixation, slides were washed in double deionized water (DDW) 3–5 times to remove all the OCT compound and stained with primary antibodies according to the manufacturer’s protocol: collagen I (1:100, Sigma-Aldrich #C2456), Collagen IV (1:100, Abcam #ab6586), Laminin (1:100, Sigma-Aldrich #L8271), Fibronectin (1:50, #ab72686), OCT-4 (1:100, Abcam #ab19857) hiPSC pluripotency marker, Cardiac troponin I (1:200, Millipore #MAB1691), Sarcomeric alpha-actinin (1:200, Abcam #ab9465), Connexin-43 (1:100, Sigma-Aldrich #C6219), CD31 (1:50, Abcam #ab28364), CD34 (1:50, Biolegend #343602), vWF (1:50, Santa Cruz #SC14014) arterial markers, Glucagon (1:200, Abcam #ab10988), and Insulin (1:100, Abcam #ab7842). For phalloidin staining, samples were similarly fixed and then stained with phalloidin-TRITC (Sigma-Aldrich) for actin fibers and with Hoechst 33258 (Sigma-Aldrich, St. Louis, MO, USA) for DNA. Images were taken using the LSM700 confocal microscope (Zeiss, Jena, Germany). Quantification was done using the Imaris software for microscopy image analysis (Oxford instruments, Oxon, UK). For each marker quantified, five images were analyzed, and the data was presented as the marker intensity, normalized to the number of cells.

### 4.6. Thermal Gravimetric Analysis (TGA)

TGA data were obtained using a TGA-Q5000 system (TA Instruments, New Castle, Delaware, USA). Lyophilized samples were heated from room temperature at a rate of 20 °C min^−1^ under a nitrogen atmosphere to a final temperature of 600°C. Data were analyzed using TA Universal Analysis Software (TA Instruments, New Castle, DE, USA).

### 4.7. Fourier Transform Infrared Spectroscopy (FTIR)

FTIR spectra were recorded using a Thermo 6700 FTIR instrument (Thermo Fisher Scientific, Waltham, MA, USA), equipped with a Smart iTR Attenuated Total Reflectance diamond plate, in the wave-number range of 500–3500 cm^−1^ (64 scans at a resolution of 4 cm^−1^). Data were evaluated using the OMNIC series software (version 8, Thermo Fisher Scientific, Waltham, MA, USA).

### 4.8. Scanning Electron Microscopy (SEM)

SEM analyses were performed using a Phenom ProX desktop SEM (PhenomWorld, Eindhoven, Netherlands), equipped with a temperature-controlled sample holder (Deben UK Ltd., Suffolk, UK). Samples were mounted on aluminum stabs using tissue freezing medium (Ted Pella, Inc., Redding, CA, USA), and cooled to −20 °C. Images were captured at 15 kV accelerating voltage.

### 4.9. Mechanical Properties of the pECM Hydrogels

Rheological analyses of the pECM hydrogels were done using a DISCOVERY HR-2 Hybrid Rheometer (TA Instruments, New Castle, DE, USA). Samples of the pre-gel solutions were transferred into the rheometer with the Peltier cell maintaining a temperature of 4 °C. The temperature was then raised to 37 °C to induce gelation and allowed to gel for 30 min. The oscillatory moduli of the sample were monitored continuously at a fixed frequency of 1 rad s^−1^ and a strain of 2.5%. When there was no further change in the elastic modulus (G’) with time, gelation was deemed to be complete. Hydrogels were tested using parallel plate geometry (40 mm diameter, 1.2 mm gap, 1% strain, 1 rad s^−1^, 37 °C). Frequencies ranged from 0.1 to 600 rad s^−1^.

### 4.10. Cell Culture

Human bone marrow mesenchymal stem cells (hMSC) passages 2–6 (Lonza, Basel, Switzerland) were cultured using αMEM, supplemented with 10% FCS, 1% Pen-Strep^®^, 0.4% Fungizone^®^, and basic fibroblast growth factor (bFGF, 5 ng ml^−1^) (Biological Industries, Beit Haemek, Israel). The media was replaced at least every 2 days. hiPSC (kindly provided by Prof. Lior Gepstein, Technion) were cultured on matrigel (Corning, Glendale, AZ, USA) in mTeSR1 Basal Medium and supplemented with mTeSR1 5X supplement (STEMCELL^TM^ Technologies, Vancouver, Canada). The medium was replaced every day. All Cells were cultured at 37 °C in a humidified incubator with 5% CO_2_.

### 4.11. Cell Culture on pECM Hydrogels

50 µL pECM pre-gel solutions, as well as alginate hydrogel (1%, *w*/*v*) and matrigel controls were allowed to gel in a 48 well tissue culture plate and kept in PBS until seeding. A total of 30,000 hMSC cells were seeded and cultured for up to 14 days. Cell viability was evaluated using the AlamarBlue™ reagent (AbD Serotec, Kidlington, UK), according to the manufacturer protocol. Cell adherence was calculated according to the cell viability one day following seeding (day 1) on the hydrogels, relative to the viability at the same time on the matrigel.

### 4.12. Hydrogel Crosslinking for Long-Term Cultures

To determine the optimal crosslinking regime for each pECM hydrogel, 40 µL of pECM hydrogel was allowed to gel in a 96 well tissue culture plate supplemented with the natural collagen crosslinkers genipin (0.1%, Sigma-Aldrich) and epigallocatechin gallate (EGCG, 0.1–1%, DSM Nutritional Products, Heerlen, Netherlands), and kept in PBS until seeding. A total of 8000 hiPSCs were seeded and cultured for 48 hr to assess cell adherence. The adherence of hiPSCs was tracked and imaged using GE InCell Analyzer 2000 at different time points, and the most appropriate crosslinking regimes were chosen. The cells were labeled using DiD (Life Technologies, Carlsbad, CA, USA) according to the manufacturer’s instructions before seeding on the hydrogels.

### 4.13. Spontaneous Differentiation of hiPSCs

100µL pECM pre-gel solutions, as well as alginate hydrogel (1%, *w*/*v*) and matrigel controls were allowed to gel in a 24 well tissue culture plate using crosslinkers as optimized (Figure S3) and kept in PBS until seeding. A total of 200,000 hiPSC were seeded and cultured for 28 days. The culture medium consisted of high-glucose DMEM supplemented with 20% serum replacement (HyClone^TM^, GE Healthcare, Chicago, IL, USA), 1% Pen-Strep^®^, 2 mM L-glutamine, 1% nonessential amino acid stock (Biological Industries, Beit Haemek, Israel), and 0.1 mM β-mercaptoethanol (Sigma-Aldrich), as previously described [67,68]. Cell viability was evaluated using the AlamarBlue™ reagent (AbD Serotec, Kidlington, UK), according to the manufacturer’s protocol. Cell adherence was calculated according to the cell viability one day following seeding (day 1) on the hydrogels, relative to the viability at the same time on the matrigel.

### 4.14. RNA Sequencing

To assess the gene expression profile of each spontaneously differentiated hiPSCs sample, after 28 days of hiPSCs culture in spontaneous differentiation conditions, whole RNA was extracted and further prepared using the CEL-Seq2 protocol compared to the original hiPSCs, with minor changes [69]. Briefly, whole RNA was extracted using Trireagent (Sigma-Aldrich). Instead of single-cells as input, 2 ng purified RNA was taken as input for library preparation. The CEL-Seq library was run on an Illumina Hiseq2500 instrument. The number of reads ranged from 2,797,839 to 20,374,324 per sample. The reads were mapped to the Homo_sapiens.GRCh38 using Tophat2 version 2.1.0, [70] with up to 2 mismatches allowed per read, the minimum and maximum intron sizes were set to 70 and 500,000, respectively, and an annotation file was provided to the mapper. The percentage of uniquely mapped reads ranged from 89.1% to 92.71% per sample. Only uniquely mapped reads were counted to genes, using ‘HTSeq-count’ package version 0.11.2 with ‘union’ mode [71]. Normalization and differential expression analyses were conducted using DESeq2 R package version 1.18.0 [72]. Sample preparation, sequencing, quality control, and differential expression analyses were conducted by the “Technion Genome Center,” Life Science and Engineering Interdisciplinary Research Center, Technion, Haifa, Israel. The DAVID bioinformatics tool (Laboratory of Human Retrovirology and Immunoinformatics, Frederick, MD, USA) as well as the Ingenuity Pathway Analysis software (IPA, QIAGEN, Aarhus C, Denmark) were used to further analyze the gene expression profiles.

### 4.15. Statistical Analysis

Unless otherwise mentioned, results are expressed as mean ± standard deviation of at least three repetitions of independent experiments per experimental group and time point. Statistical differences between means were determined using a *t*-test for individual comparisons or by two-way ANOVA when appropriate, with the Holm-Sidak method or Tukey’s multiple comparisons test, respectively. Statistical significance was defined as *p* < 0.05 unless otherwise mentioned.

## 5. Conclusions

The great potential of pECM-derived hydrogels as promising biomaterials for numerous biomedical applications was established in recent years, thus emphasizing the biocompatible and bioactive properties of these materials, which support cellular growth and recruitment. In the present work, however, we show that a critical consideration in designing such an application should be the tissue of origin from which the pECM is derived. Hence, the unique composition of each pECM hydrogel dictates the constitution of a different hydrogel microstructure which is characterized by different mechanical properties. These differences, in turn, lead to the establishment of a tissue-specific microenvironment, in which the reciprocal communication with the residing cells is exclusive to each tissue-derived pECM hydrogel. Accordingly, the cell’s adherence, survival, proliferation, and function highly depend on the type of pECM hydrogel on which they are cultured. Furthermore, when culturing pluripotent stem cells, their spontaneous differentiation is directed by the matrix towards the lineages which characterize the pECM original tissue. The translational implications of these findings can include guiding future developments of biomedical platforms to use tissue-specific ECM hydrogels (e.g., pancreas pECM hydrogel for an artificial pancreas) but also to induce desired processes where they are needed (e.g., arterial pECM hydrogel to induce angiogenesis).

## Figures and Tables

**Table 1:** Decellularized pECM concentration for hydrogel gelation.

Source Organ	Minimal pECM Concentration for Gelation (mg ml^−1^)
Cardiac	10
Artery	30
Pancreas	20
Adipose	10

**Table 2:** Thermal gravimetric analysis summary of the degradation steps.

Sample	Stage	$T_{\mathrm{Onset}}\;{}^{\circ}C$	$T_{\mathrm{Peak}}\;{}^{\circ}C$	$T_{\mathrm{Enset}}\;{}^{\circ}C$	Weight (%)	Residue (%)
Cardiac ECM hydrogel	I	33.0±3.2	47.9±5.3 *	108.7±6.7 *	7.5±3.1	22.7±3.8
	II	128.8±2.9	156.6±0.5	187.7±1.8	5.2±2.5	
	III	236.4±5.1	294.4±10.0	508.4±29.1 *	44.2±12.1 *	
Artery ECM hydrogel	I	39.0±1.3	71.9±10.7 *	133.1±3.3 *	10.2±0.2	19.8±1.1
	II	144.6±2.7	156.1±1.1 *	175±1.0	2±0.5	
	III	226.5±2.6 *	297.2±5.9	480.2±16.5 *	65.9±1.1 *	
Pancreas ECM hydrogel	I	46.0±7.5	56.1±5.9	102.8±9.7 *	3.7±1.3	29.4±1.9
	II	141.0±12.5	188.7±6.2 *	227.3±5.0 *	15.3±0.7	
	III	252.7±12.6 *	294.6±3.8	488.1±11.4	30.3±6.0 *	
Adipose ECM hydrogel	I	39.2±4.5	46.8±5.1 *	112.2±0.3 *	9.8±0.7	24.7±0.9
	II	133.3±0.3	155.8±0.4 *	183.7±1.8 *	2.6±0.1	
	III	242.5±1.0	301.1±1.3	491.3±1.2	56.8±0.6 *	

* *p* < 0.001.

## Data Availability

The data presented in this study are available on request from the corresponding author.

## Supplementary Materials

The following are available online at https://www.mdpi.com/article/10.3390/ijms222111624/s1. Figure S1: Thermal gravimetric analysis of pECM hydrogels and decellularized pECM. Figure S2: Preservation of the molecular structure of pECM hydrogels. Figure S3: hiPSCs adherence to crosslinked pECM hydrogels. Figure S4: Spontaneous differentiation of hiPSCs on pECM hydrogels. Figure S5: RNA-seq of spontaneously differentiated hiPSCs on pECM hydrogels.
