# Supplementary material for: Extracellular Matrix Hydrogels Originated from Different Organs Mediate Tissue-Specific Properties and Function
Source: Int J Mol Sci. 2021 Oct 27;22(21):11624. doi: 10.3390/ijms222111624 (PMC8583810; doi:10.3390/ijms222111624)
Supplement: Supplementary file 1 [file ijms-22-11624-s001.zip › ijms-1398049-supplementary.pdf]

Supplementary Information

Extracellular Matrix Hydrogels Originated from Different Organs mediate Tissue-Specific Properties and Function

Tzila Davidov, Yael Efraim, Rotem Hayam, Jacopo Oieni, Limor Baruch, and Marcelle Machluf*

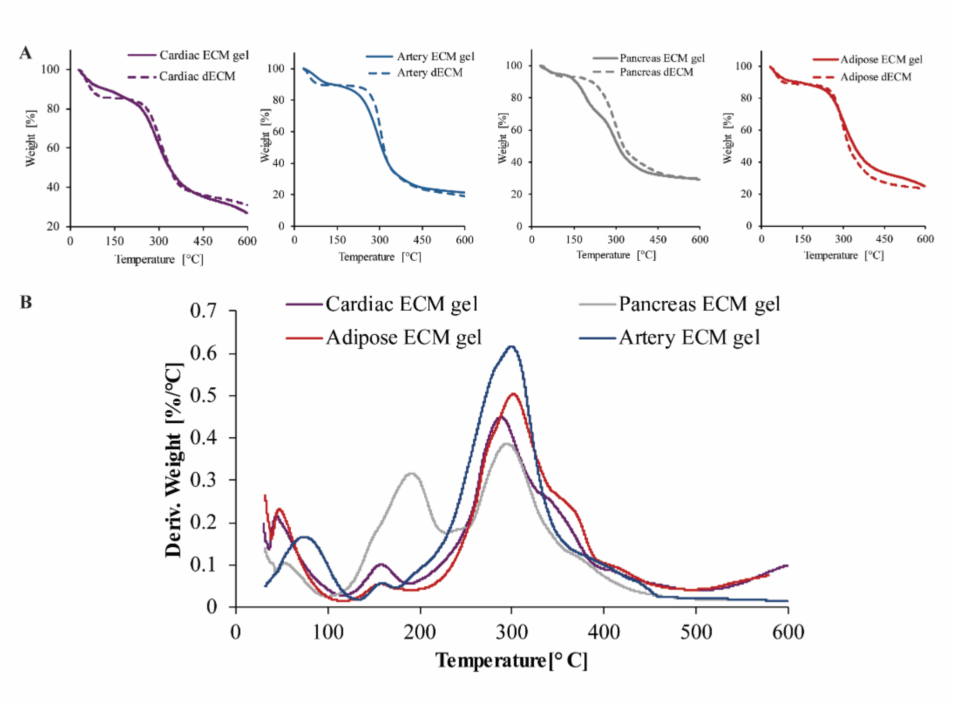

Figure S1. **Thermal gravimetric analysis of pECM hydrogels and decellularized pECM**. Samples weight (A) and their 1st derivative (B) upon increasing the temperature of cardiac, artery, pancreas, and adipose pECM.

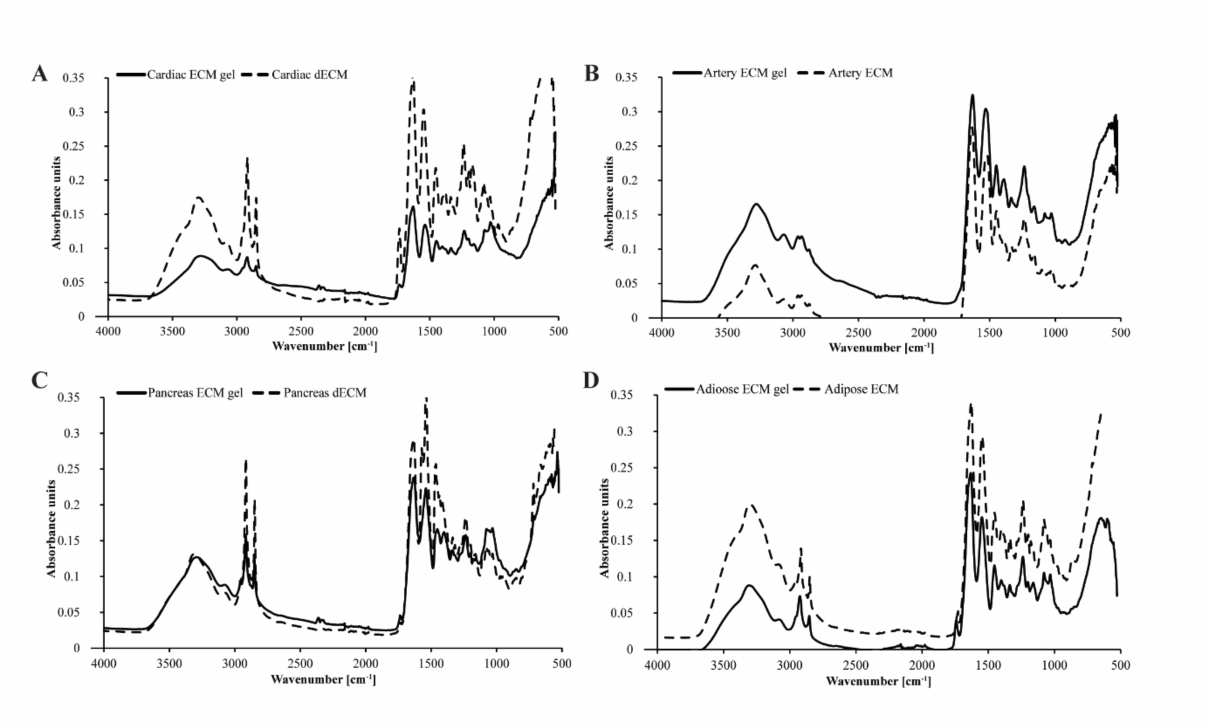

Figure S2. **Preservation of the molecular structure of pECM hydrogels**. FTIR analysis of pECM hydrogels and decellularized pECM of (A) cardiac, (B) artery, (C) pancreas, and (D) adipose pECM.

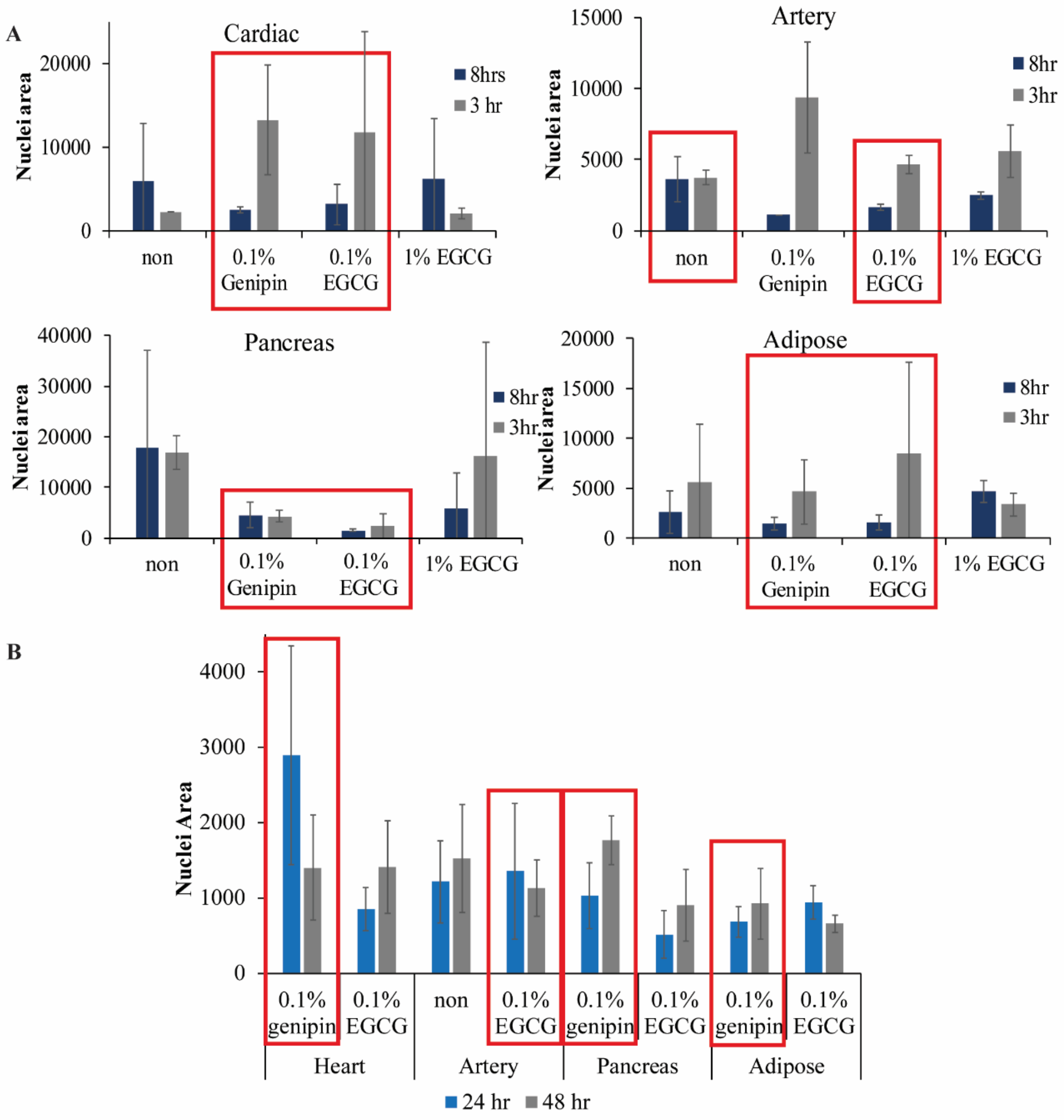

Figure S3. **hiPSCs adherence to crosslinked pECM hydrogels**. InCell 2000 quantitative analysis determining the hiPSCs adherence to pECM hydrogels supplemented with natural crosslinkers. Cell adherence is represented by the nuclei area. (A) Cell adherence 3 and 8 hours post-seeding using variable crosslinkers, and (B) 24 and 48 hours post-seeding using the chosen crosslinking conditions.

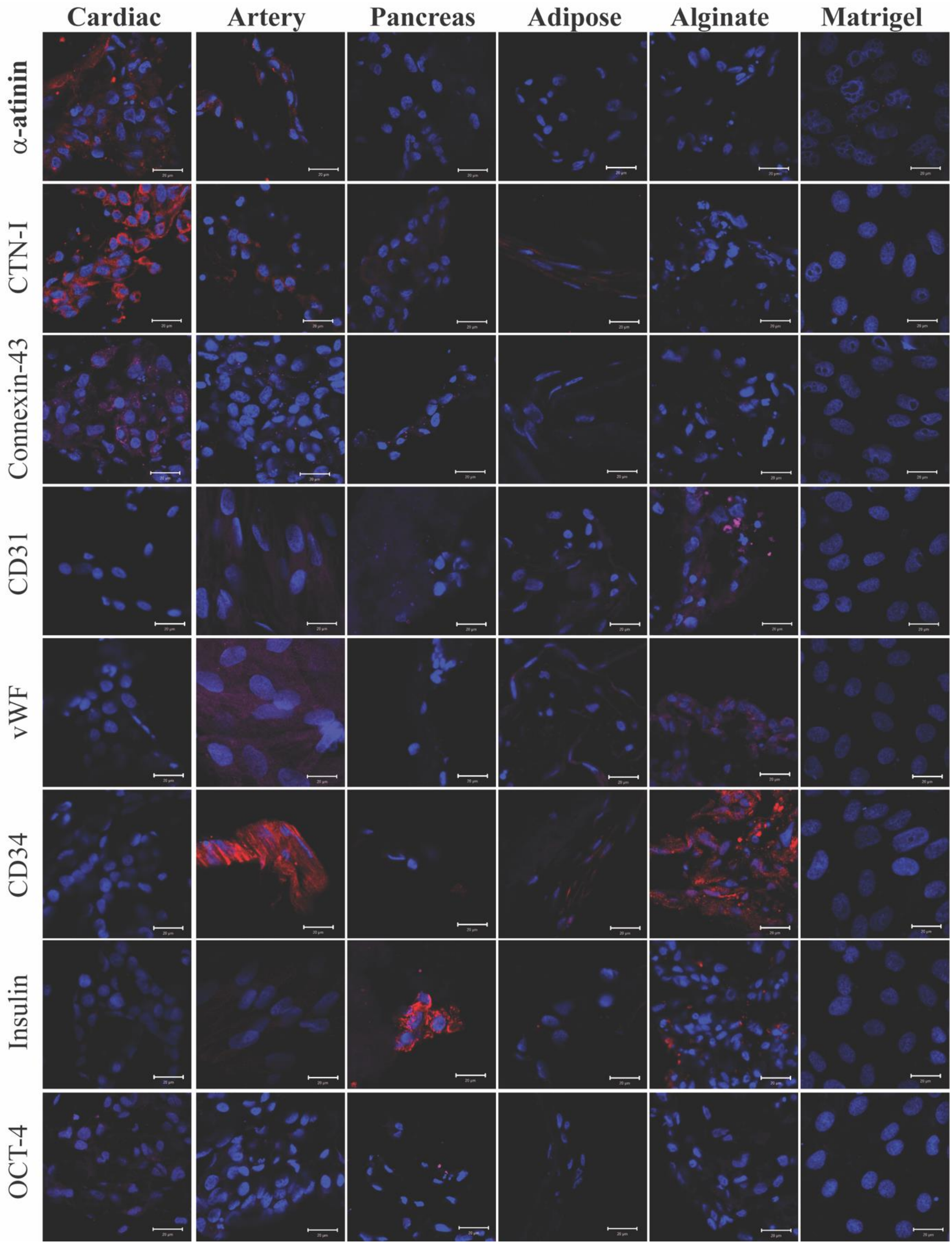

Figure S4. **Spontaneous differentiation of hiPSCs on pECM hydrogels**. Immunostaining of spontaneously differentiated hiPSCs 28 days post-seeding. Red: α-actinin, cTnI, CD34, and Insulin. Magenta: connexin-43, CD31, vWF and OCT-4. Blue: DAPI (DNA). Scale bars, 20 µm.

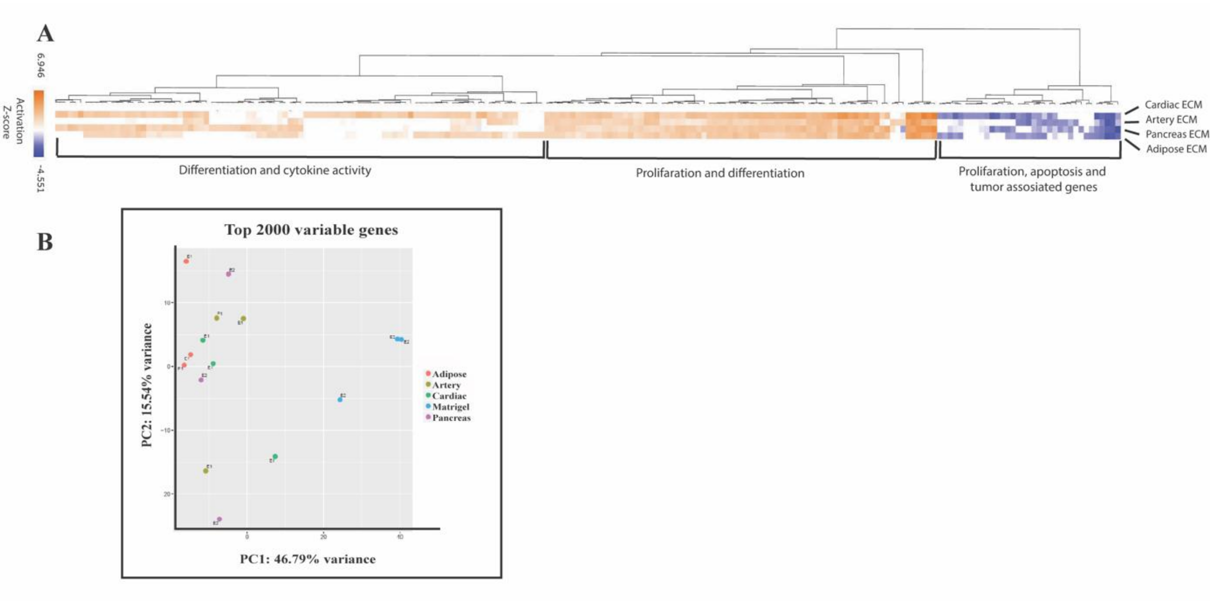

Figure S5. **RNA-seq of spontaneously differentiated hiPSCs on pECM hydrogels**. (A) Hierarchical clustering heat map of gene expression by hiPSCs cultured on the different pECM hydrogels relative to hiPSCs cultured on matrigel. Relative expression (z-score) is displayed in colors, in which orange indicates upregulation and blue indicates downregulation. (B) Principal component analysis (PCA) of the top 2000 variable genes. A comparison between cells cultured on all pECM hydrogels and matrigel.
